# Fluorescence-Based Flow Sorting in Parallel with Transposon Insertion Site Sequencing Identifies Multidrug Efflux Systems in *Acinetobacter baumannii*

**DOI:** 10.1128/mBio.01200-16

**Published:** 2016-09-06

**Authors:** Karl A. Hassan, Amy K. Cain, TaoTao Huang, Qi Liu, Liam D. H. Elbourne, Christine J. Boinett, Anthony J. Brzoska, Liping Li, Martin Ostrowski, Nguyen Thi Khanh Nhu, Tran Do Hoang Nhu, Stephen Baker, Julian Parkhill, Ian T. Paulsen

**Affiliations:** a Department of Chemistry and Biomolecular Sciences, Macquarie University, Sydney, NSW, Australia; b Wellcome Trust Sanger Institute, Hinxton, Cambridge, United Kingdom; c Liverpool School of Tropical Medicine, Malawi-Liverpool-Wellcome Trust Clinical Research Programme, Blantyre, Malawi; d The Hospital for Tropical Diseases, Wellcome Trust Major Overseas Programme, Oxford University Clinical Research Unit, Ho Chi Minh City, Vietnam; e School of Chemistry and Molecular Biosciences, The University of Queensland, Brisbane, Queensland, Australia; f Centre for Tropical Medicine, Nuffield Department of Clinical Medicine, Oxford University, Oxford, United Kingdom

## Abstract

Multidrug efflux pumps provide clinically significant levels of drug resistance in a number of Gram-negative hospital-acquired pathogens. These pathogens frequently carry dozens of genes encoding putative multidrug efflux pumps. However, it can be difficult to determine how many of these pumps actually mediate antimicrobial efflux, and it can be even more challenging to identify the regulatory proteins that control expression of these pumps. In this study, we developed an innovative high-throughput screening method, combining transposon insertion sequencing and cell sorting methods (TraDISort), to identify the genes encoding major multidrug efflux pumps, regulators, and other factors that may affect the permeation of antimicrobials, using the nosocomial pathogen *Acinetobacter baumannii*. A dense library of more than 100,000 unique transposon insertion mutants was treated with ethidium bromide, a common substrate of multidrug efflux pumps that is differentially fluorescent inside and outside the bacterial cytoplasm. Populations of cells displaying aberrant accumulations of ethidium were physically enriched using fluorescence-activated cell sorting, and the genomic locations of transposon insertions within these strains were determined using transposon-directed insertion sequencing. The relative abundance of mutants in the input pool compared to the selected mutant pools indicated that the AdeABC, AdeIJK, and AmvA efflux pumps are the major ethidium efflux systems in *A. baumannii*. Furthermore, the method identified a new transcriptional regulator that controls expression of *amvA*. In addition to the identification of efflux pumps and their regulators, TraDISort identified genes that are likely to control cell division, cell morphology, or aggregation in *A. baumannii*.

## IMPORTANCE

Transposon-directed insertion sequencing (TraDIS) and related technologies have emerged as powerful methods to identify genes required for bacterial survival or competitive fitness under various selective conditions. We applied fluorescence-activated cell sorting (FACS) to physically enrich for phenotypes of interest within a mutant population prior to TraDIS. To our knowledge, this is the first time that a physical selection method has been applied in parallel with TraDIS rather than a fitness-induced selection. The results demonstrate the feasibility of this combined approach to generate significant results and highlight the major multidrug efflux pumps encoded in an important pathogen. This FACS-based approach, TraDISort, could have a range of future applications, including the characterization of efflux pump inhibitors, the identification of regulatory factors controlling gene or protein expression using fluorescent reporters, and the identification of genes involved in cell replication, morphology, and aggregation.

## Observation

To be effective in killing or stalling the growth of bacterial cells, antimicrobials must reach their cellular targets. For the majority of antimicrobials, these targets are in the cytoplasm, meaning that they must cross the cell envelope to induce their effects. The cell envelope is a particularly important factor for antimicrobial resistance in Gram-negative bacteria, since it includes two membrane permeability barriers with different surface chemistries, presenting significant potential to limit the accumulation of chemically diverse antimicrobial compounds (1).

In addition to preventing accumulation of antimicrobials, all bacteria employ sets of efflux pumps that mediate the active expulsion of these compounds should they cross a biological membrane (2). Many antimicrobial efflux pumps in bacteria have multidrug recognition profiles. Therefore, the increased expression of a single pump can result in resistance to a broad spectrum of antimicrobial classes. In Gram-negative bacteria, efflux pump overexpression has been shown to promote clinically significant levels of antimicrobial resistance (3). Genes encoding efflux pumps have been identified in all bacterial genomes sequenced to date and can be found in large numbers (4). For example, strains of the opportunistic human pathogen *Acinetobacter baumannii* typically encode more than 50 putative efflux pumps, accounting for approximately 1.5% of their protein coding potential (5).

Despite their abundance, only a few transporters resembling drug efflux pumps have been experimentally characterized in most bacterial species. It can be difficult to discern which, if any, of the uncharacterized pumps could play an active role in protecting the cell against cytotoxic compounds without conducting labor-intensive experimental investigations. Furthermore, it can be even more challenging to identify the regulatory proteins that control expression of active multidrug efflux pumps. In this study, we sought to identify these proteins in *A. baumannii* by directly assessing drug accumulation within a population of more than 100,000 random transposon mutants. To this end, we applied fluorescence-activated cell sorting (FACS) in parallel with transposon-directed insertion sequencing (TraDIS) (6, 7). This novel approach, which we have named “TraDISort,” was able to identify genes in *A. baumannii* that are associated with increased or decreased accumulation of ethidium bromide, a cationic quaternary ammonium derivative and a common substrate of multidrug efflux pumps.

### Fluorescence-activated cell sorting to enrich for mutants displaying aberrant accumulation of ethidium.

Ethidium readily intercalates into nucleic acids, whereupon its fluorescence intensity increases significantly. Consequently, ethidium is differentially fluorescent inside and outside cells, and cellular fluorescence can be used as a proxy for its cytoplasmic concentration (8). We hypothesized that when cells are treated with a subinhibitory concentration of ethidium, the ethidium concentrations in the cytoplasm of cells with defective multidrug efflux machinery should be higher than the concentration in wild-type cells at equilibrium, and conversely, the concentration in cells with overactive efflux machinery should be below that in wild-type cells To test this hypothesis, we examined populations of three isogenic strains of *A. baumannii* AB5057-UW (9) that differentially expressed AdeIJK, a major multidrug efflux pump in *A. baumannii*, which recognizes ethidium as a substrate (10, 11): (i) wild-type AB5075-UW, (ii) a mutant containing a transposon insertion in *adeJ*, and (iii) a mutant containing a transposon insertion in *adeN*, which encodes a negative regulator of *adeIJK* expression (9). When examined by flow cytometry, populations of the different cell types displayed distinct but partially overlapping fluorescence profiles that were in agreement with our predictions, i.e., the average fluorescence of the *adeJ* and *adeN* mutant populations was above and below that of the wild-type population, respectively (see Fig. S1A in the supplemental material). We repeated this experiment, using equivalent isogenic strains of *Acinetobacter baylyi* ADP1 (5), and made the same observations (see Fig. S1B). Based on these experiments, we predicted that it would be possible to use FACS to enrich cells from a large mutant pool that display differential ethidium accumulation or efflux based on their fluorescence intensity.

A mutant library containing more than 100,000 unique insertion mutants of *A. baumannii* BAL062 was generated using a Tn*5*-based custom transposon, and the insertion sites in the mutant pool were mapped by TraDIS (7). This library was treated with 40 µM ethidium bromide (1/16× MIC of the parental strain) and subjected to FACS to collect cells containing the highest concentrations of ethidium (i.e., the 2% most fluorescent cells) and cells containing the lowest concentrations of ethidium (i.e., the 2% least fluorescent cells). DNA was isolated from the selected pools of cells, and TraDIS was used to identify the chromosomal locations of the Tn*5* insertion sites in these cells (7). Transposon insertions were significantly (>2-fold change; *Q* value, <0.05) less abundant in 162 genes and more abundant in 24 genes in the low-fluorescence population and less abundant in 159 genes and more abundant in 24 genes in the high-fluorescence population compared to the input pool (see Data Set S1 in the supplemental material).

### FACS in parallel with TraDIS identifies the active ethidium efflux pumps encoded by *A. baumannii* and core efflux pump regulators.

**FIG 1:**
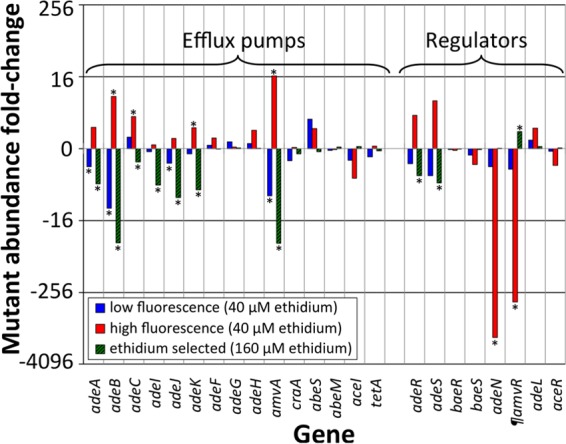
Selection of *A. baumannii* mutants carrying insertions in genes encoding the characterized efflux pumps AdeABC (12), AdeIJK (10), AdeFGH (20), AmvA (13, 14), CraA (21), AbeS (22), AbeM (23), and AceI (24, 25) and regulators AdeRS and BaeRS, which control expression of *adeABC* (15, 26); AdeN, which controls *adeIJK* (16); AdeL, which controls *adeFGH* (20); and AceR, which controls *aceI* (27). Bars represent the fold change in mutant abundance in cells selected for low ethidium fluorescence (blue), high ethidium fluorescence (red), or growth in 62.5 µg/ml (approximately 158 µM) ethidium bromide (hatched green; 1/4× MIC) compared to the starting mutant pool. Positive values indicate higher mutant abundance in the selected pool, whereas negative values indicate lower abundance. Asterisks indicate values supported by a *Q* value of 0.05 or below. ¶, the gene named here as *amvR* encodes a TetR family regulator that represses *amvA* gene expression (see text for details).

Following the experiments with targeted mutants, we hypothesized that many cells containing the highest concentrations of ethidium would have transposon insertions in genes encoding efflux pumps or activators of efflux pumps, and conversely, cells containing the lowest concentrations of ethidium would have insertions in genes encoding negative regulators of efflux pumps. Comparisons of the insertion sites in the mutant input pool with those in the high- and low-fluorescence pools supported this proposal (Fig. 1; see also Data Set S1 in the supplemental material). Mutants carrying insertions in genes encoding several multidrug efflux pumps, particularly *adeABC* (12), *adeIJK* (10), and *amvA* (13, 14), and genes encoding the *adeABC* activator, *adeRS* (15), were overrepresented in the highly fluorescent populations (Fig. 1). Inactivation of these genes is likely to reduce the rate of efflux and thus result in a higher cytosolic concentration of ethidium. In contrast, inactivated mutants of these genes were less abundant in the low-fluorescence populations (Fig. 1), since the efflux pumps encoded or regulated by these genes help to lower the concentration of ethidium in the cell. We used the Transporter Automated Annotation Pipeline (http://www.membranetransport.org/) to search for genes encoding novel efflux pumps in the *A. baumannii* BAL062 genome. We identified 56 genes that are likely to encode novel efflux pumps, or components of novel efflux pumps, based on their primary sequence characteristics (see Table S1 in the supplemental material). These efflux pumps are likely to recognize small-molecule substrates, but our data did not suggest that any of these efflux pumps have a significant *in vivo* role in ethidium efflux, since none were significantly differentially selected by our fluorescence-based selection (see Table S1).

Some of the most highly differentially selected genes in the flow-sorted samples were genes that encode transcriptional repressors known or predicted to control expression of multidrug efflux systems. For example, mutants carrying insertions in *adeN*, which controls expression of *adeIJK* (16), were 1,469-fold less abundant in the highly fluorescent output pool compared to the input pool (Fig. 1). Additionally, mutants carrying insertions in BAL062_01495, which encodes a TetR family regulator, were 371-fold less abundant in the highly fluorescent output pool compared to the input pool (Fig. 1). BAL062_01495 is adjacent to and divergently transcribed from *amvA* in the BAL062 chromosome. To test whether the TetR family protein encoded by BAL062_01495 was able to regulate expression of *amvA*, we compared *amvA* expression levels in the *A. baumannii* AB5075-UW parental strain and a strain harboring a transposon insertion in the gene orthologous to BAL062_01495. The level of *amvA* expression measured by reverse transcription-quantitative PCR (qRT-PCR) (5) in the mutant strain was 5.7- ± 1.9-fold higher than that in the parental strain during late exponential phase, indicating that the TetR family regulator controls expression of *amvA*. Consequently, we have tentatively named this novel regulator AmvR.

**FIG 2:**
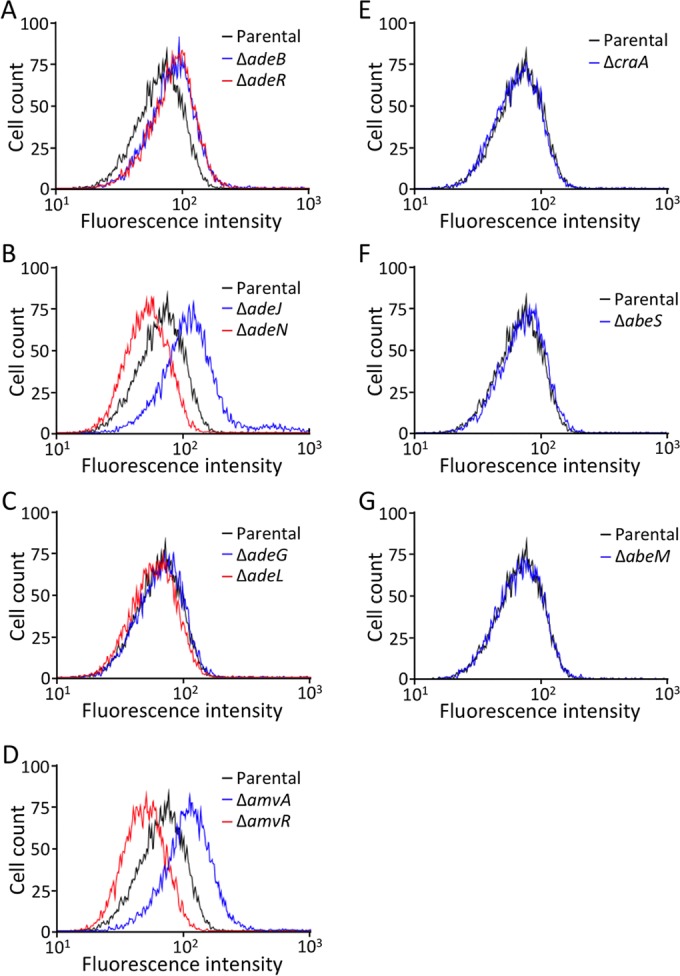
Flow cytometric analysis of *Acinetobacter baumannii* AB5075-UW parental strain (black), inactivated efflux pump mutants (blue), and inactivated efflux regulatory mutants (red). The fluorescence profile of the parental population is shown in all panels and is overlaid with the profiles of Δ*adeB* and Δ*adeR* (A), Δ*adeJ* and Δ*adeN* (B), Δ*adeG* and Δ*adeL* (C), Δ*amvA* and Δ*amvR* (D), Δ*craA* (E), Δ*abeS* (F), and Δ*abeM* (G). Cell populations were exposed to 40 µM ethidium bromide, and each curve shows the fluorescence intensity for 10,000 cells. The cell populations show distinct fluorescence profiles based on the concentration of ethidium in the cell cytoplasm.

To confirm the specific involvement of different multidrug efflux pumps and their regulators in controlling the accumulation of ethidium in *A. baumannii*, we conducted flow cytometry on targeted mutants of *adeB*, *adeR*, *adeJ*, *adeN*, *adeG*, *adeL*, *amvA*, *amvR*, *craA*, *abeS*, and *abeM*. These mutant strains were loaded with 40 µM ethidium bromide, and the fluorescence of 10,000 cells was determined by flow cytometry (Fig. 2). The TraDISort method identified the AdeABC, AdeIJK, and AmvA efflux systems and their regulators, AdeRS, AdeN, and AmvR, as playing a role in ethidium accumulation. The fluorescence profiles of the specific mutant populations closely reflected these findings. As seen in our preliminary experiments (see Fig. S1 in the supplemental material), the average fluorescence of the *adeJ* and *adeN* mutant populations was above and below that of the parental cell population, respectively (Fig. 2B). The *amvA* and *amvR* mutant cells showed fluorescence profiles very similar to those of *adeJ* and *adeN* mutants, respectively (Fig. 2D), in line with the function of AmvR as a repressor of *amvA* expression. The average fluorescence of the *adeB* and *adeR* mutant cell populations was a similar degree higher than that of the parental population, highlighting the role of AdeB in ethidium efflux and of AdeR in controlling the expression of *adeABC* (Fig. 2A). The fluorescence profiles of mutant populations of other multidrug efflux systems, which were not identified using the TraDISort approach, were very similar to that of the parental strain (Fig. 2).

### TraDIS following fitness-induced selection using ethidium bromide.

In addition to FACS to enrich for cells displaying aberrant accumulation of ethidium, we cultured the mutant library in the presence of ethidium bromide. This experiment used a higher concentration of ethidium bromide (1/4× MIC of the parental strain) than that used in the FACS analyses to impose a chemical selection that would allow us to identify mutants with a fitness advantage or defect in the presence of ethidium by TraDIS. In the ethidium bromide-selected mutant pools, transposon insertions were less abundant in 63 genes and more abundant in eight genes compared with the input control pools. This suggests that gene loss generally results in a fitness defect, rather than advantage, under ethidium selection, which is in keeping with general evolutionary theory. Mutants containing transposon insertions in efflux pump genes and their regulators were the most highly differentially selected by ethidium bromide. The pattern of selection among these mutants overlapped with the selection pattern in the low-ethidium-fluorescence FACS experiment (Fig. 1). For example, mutants carrying transposon insertions in the *adeABC*, *adeIJK*, *amvA*, and *adeRS* genes were less abundant in the ethidium-selected output pool (Fig. 1), confirming the role of these multidrug efflux pumps and regulators in resistance to ethidium.

Similarly to efflux pump genes and their regulators, mutations in the DNA modification methylase gene, BAL062_03687, were significantly negatively selected by ethidium bromide and less abundant in the low-fluorescence samples compared to the input pool. Methylation mediated by BAL062_03687 could protect DNA from ethidium intercalation and thereby reduce fluorescence and provide resistance to ethidium-induced mutation.

Several genes controlling the composition of the cell membranes, cell wall, or capsule were also negatively selected by ethidium bromide (BAL062_00585, BAL062_00596, BAL062_01038, BAL062_03374, BAL062_03418, BAL062_03480, BAL062_03481, BAL062_03674, and BAL062_03869 [see Data Set S1 in the supplemental material]). These genes may help to reduce uptake of ethidium. Some of these genes were significantly negatively selected in both the low- and high-fluorescence FACS-selected samples and could thus play a role in controlling cell morphology or size (see below). In contrast, several capsule biosynthesis genes (BAL062_03853, BAL062_03857, and BAL062_03858) were positively selected by the ethidium treatment. This highlights the influence that the sugar composition of the capsule could play in regulating the accumulation of amphipathic small molecules into the cell.

### FACS in parallel with TraDIS identifies genes involved in cell division and aggregation.

In conducting FACS to enrich for mutants displaying aberrant accumulation of ethidium in *A. baumannii*, we gated to target cells with uniform forward and side scatter and limited the collection of dead or aggregated cells that may complicate downstream analyses (see Fig. S2 in the supplemental material). As a consequence of this gating, we identified a number of mutants that are likely to have cell division defects or enhanced aggregation properties. These mutants were negatively selected in both the low- and high-fluorescence FACS-selected pools relative to the input pool, and included 80 (49.4 to 50.3%) of the significantly selected genes in these pools. For example, mutants carrying insertions in the *mreBCD* gene cluster (BAL062_00713 to BAL062_00715), *rlpA* (BAL062_01224), *rodA* (BAL062_01226), and *ftsI* (BAL062_02811), which are likely to function in cell division, were in very low abundance in each of the flow-sorted mutant pools relative to the input pool (see Fig. S3). Mutants carrying insertions in biotin biosynthesis genes were also significantly less abundant in the FACS-selected pools than in the input pool and, to a lesser extent, in the ethidium-selected pools. The role of biotin in ethidium resistance, cell structure, or aggregation is at present unknown but may be related to its function as a cofactor in fatty acid synthesis. Two capsular polysaccharide biosynthetic genes were significantly less abundant in both of the flow-sorted mutant pools than in the input pool. These mutants may have a higher tendency toward aggregation or different cell morphologies or may display light-scattering properties different from those of other mutant cells (see Fig. S3). Approximately 20% of the inactivated genes in mutants negatively selected by FACS were annotated as hypothetical proteins, and many more had been assigned only putative functions. These genes could be targeted in future investigations exploring cell division and aggregation/biofilm formation in *A. baumannii*.

While insertions in genes implicated in cell replication and increased aggregation were negatively selected by the flow sorting, there appeared to be enrichment for mutants that are less likely to aggregate in culture. The majority of these mutants harbored transposon insertions in the *csu* type I pilus biosynthesis and regulatory gene cluster (BAL062_01328 to BAL062_01334 [see Fig. S4 in the supplemental material]). These genes are likely to function in biotic or abiotic cell adherence/aggregation and biofilm formation (17). Therefore, we suspect that the strains carrying mutations in these genes are less likely to aggregate, leading to their enrichment in our flow-sorted samples.

### Conclusions.

In this study, we identified the genes that control accumulation of the antimicrobial dye ethidium into the Gram-negative hospital-associated pathogen *A. baumannii*. We exploited the differential fluorescence of ethidium inside and outside the cell to enrich for mutants showing aberrant accumulation of ethidium by FACS and used TraDIS to identify the transposon insertion sites within the enriched mutants. This work highlighted the importance of three multidrug efflux systems, AdeABC, AdeIJK, and AmvA, in reducing ethidium accumulation and promoting resistance. We also confirmed the importance of two regulatory systems, AdeRS and AdeN, that control expression of two of these pumps and identified the first known regulator for the AmvA efflux pump, which we have called AmvR. These results demonstrate the utility of the TraDISort method in identifying bacterial multidrug resistance efflux pumps and will be particularly useful when studying bacterial species for which little is known with respect to the major efflux systems. In addition to the core efflux pumps, the TraDISort method identified a large number of novel genes that are likely to be involved in cell division and/or aggregation. This application considerably expands the scope of utility for this method.

To our knowledge, this study represents the first time that FACS or any other physical selection method has been applied in parallel with TraDIS to physically enrich for phenotypes of interest in mutant populations prior to sequencing. The results demonstrate the feasibility of this combined approach to generate statistically significant results and avoid potential false positives that can arise in traditional fluorescent screening approaches, where individual strains are isolated and studied. In addition to those applications described above, we anticipate that FACS applied in parallel with TraDIS could have a range of additional applications in microbiological research: for example, to rapidly screen saturation mutant libraries carrying fluorescent reporters for genes involved in regulation, to identify the efflux pumps inhibited by novel efflux inhibitors, and to inform *in vitro* evolution studies with fluorescent reporters to identify mutants with improved metabolic productivity (18).

### Ethidium accumulation in isogenic *Acinetobacter* mutants measured by flow cytometry.

*Acinetobacter baumannii* AB5075-UW and Tn*26* insertion mutants of *adeB* (ABUW_1975-150::T26), *adeR* (ABUW_1973-195::T26), *adeJ* (ABUW_0843-122::T26), *adeN* (ABUW_1731-148::T26) *adeG* (ABUW_1335-195::T26), *adeL* (ABUW_1338-193::T26), *amvA* (ABUW_1679-169::T26), *amvR* (ABUW_1678-136::T26), *craA* (ABUW_0337-173::T26), *abeS* (ABUW_1343-187::T101), and *abeM* (ABUW_3486-184::T26) were obtained from the Manoil lab collection (9). The strains were grown in Mueller-Hinton (MH; Oxoid) broth with shaking overnight, diluted 1:100 in fresh MH broth, grown to late exponential phase, and diluted to an optical density at 600 nm (OD_600_) of 0.6 in MH broth containing 40 µM ethidium bromide (Sigma-Aldrich), approximately 1/16 of the MIC for the parental strain (250 µg/ml). This concentration is below the MIC for all strains tested and provided good fluorescent resolution between cells differentially expressing an efflux pump. The cells were incubated at room temperature for 20 min and then further diluted to an OD_600_ of 0.018 in MH broth containing 40 µM ethidium bromide for flow cytometric analyses. The ethidium fluorescence of 10,000 cells from each population was examined on a BD Influx flow cytometer using a 200-mW 488-nm laser (Coherent Sapphire) equipped with a small particle forward scatter detector. Ethidium bromide fluorescence was detected using a 580/30 bandpass filter. The cells were counted from within populations gated by forward scatter versus forward scatter pulse width, to discriminate against aggregated cells, followed by forward and side scatter to ensure that only living cells of uniform size were examined (see Fig. S2 in the supplemental material). *Acinetobacter baylyi* ADP1 wild type and *adeJ* and *adeN* mutants, generated in our previous studies (5), were examined according to the same method, except that 15 µM ethidium bromide was used due to the higher susceptibility of this strain to ethidium.

### Transposon mutant library generation and verification by TraDIS.

A dense Tn*5* mutant library was constructed in *A. baumannii* BAL062, a global clone II isolate (ENA accession numbers LT594095 to LT594096), as previously described (6, 7). Briefly, a custom transposome that included a kanamycin resistance cassette amplified from the pUT-km1 plasmid was generated using the EZ-Tn5 custom transposome construction kit (Epicentre). The custom transposome was electroporated into BAL062, and the cells were plated on kanamycin selective medium (10 mg/liter). More than 100,000 mutants were collected and stored as glycerol stocks at −80°C. Aliquots of stock containing approximately 10^9^ cells were grown overnight in MH broth. Genomic DNA was isolated from the cultures, and the transposon insertion sites were sequenced across four lanes of the Illumina HiSeq sequencing system. The insertion sites were mapped and analyzed statistically using protocols and bioinformatic tools in the TraDIS toolkit (7). The number of insertions per gene, as a factor of gene size (insertion index), was calculated for cells grown in MH broth to illustrate the evenness of transposon insertions across the genome and to show that the library was sufficiently saturated for experimental analyses. Insertions at the extreme 3′ end (last 10%) of each gene were filtered since they may not inactivate the gene. When the data were plotted against frequency, we observed a bimodal distribution of insertion indexes in the BAL062 library, with the peaks correlating with genes that tolerate or do not tolerate insertions when cultured under permissive growth conditions (see Fig. S5 in the supplemental material) (19). Using the method described in reference 20, as executed through the TraDIS toolbox (7), essential genes were identified as those with an insertion index below 0.0047 (*n* = 475) and were excluded from later analyses (see Fig. S5). On average, among the nonessential genes (*n* = 3,362) there were 35.9 unique insertions per kb of gene sequence (see Fig. S5).

### FACS to enrich for *A. baumannii* mutants showing aberrant accumulation of ethidium.

An aliquot of BAL062 mutant library stock containing approximately 10^9^ cells was grown overnight in MH broth. The overnight culture was diluted 1:100 and grown to late exponential phase (OD_600_ of 5.5). The cells were diluted to an OD_600_ of 0.6 in MH broth containing 40 µM ethidium bromide (approximately 1/16 of the MIC of the parental strain) and then further diluted 1:100 in 40 µM ethidium bromide for FACS. This concentration of ethidium bromide was used because it provided excellent differentiation between mutants known to differentially accumulate ethidium (see Fig. S1 in the supplemental material) and was well below the MIC of these mutants, so that it would not cause changes to the mutant ratios because of cell death during the sorting procedure. Cells were sorted using a BD Influx flow cytometer on the basis of ethidium fluorescence (as described above) using the highest purity mode (1 drop single). Single cells with uniform forward and side scatter were gated, and pools of the most highly and weakly fluorescent cells (2% of total single cells) within this gate were collected in separate tubes containing fresh MH broth (150,000 to 175,000 cells across four replicates [see Table S3 in the supplemental material]). The cells collected were grown overnight, DNA was isolated, and insertion sites were mapped by TraDIS as described above. Comparisons between ratios of insertion sites in the control and experimental mutant pools were made using the statistical comparison scripts in the TraDIS toolbox (7). Genes with fewer than 10 mapped reads in any data set being compared were excluded from the analyses. Genes described as being significantly differentially selected between the control and experimental samples were those showing a greater than 2-fold change in mutant abundance with a *Q* value below 0.05.

For comparison to the FACS-enriched mutants, we also selected mutants based on their competitive fitness in ethidium bromide. An aliquot of BAL062 mutant library stock containing approximately 10^9^ cells was grown overnight in MH broth. The overnight culture was diluted 1:100 and grown overnight in 62.5 µg/ml (158.5 µM) of ethidium bromide (equivalent to 1/4 of the MIC for the parental strain) to impose a chemical selection that would allow us to identify mutants with a fitness advantage or defect in the presence of ethidium bromide. Genomic DNA was isolated, and the insertion sites were determined by TraDIS. A replicate experiment with no ethidium was used as the reference in these experiments.

### Accession number(s).

The TraDIS sequence data files were deposited into the European Nucleotide Archive under accession numbers listed in Table S2 in the supplemental material.

## SUPPLEMENTAL MATERIAL

Figure S1  Flow cytometric analysis of *Acinetobacter* parental (black), and Δ*adeN* (red) and Δ*adeJ* (blue) mutant populations exposed to ethidium bromide. (A) *A. baumannii* AB5075-UW parental and mutant populations exposed to 40 µM ethidium bromide. The AB5075-UW strains were obtained from the Manoil lab collection (9). The mutants carry Tn*26* insertions in *adeJ* (ABUW_0843-122::Tn*26*) and *adeN* (ABUW_1731-148::Tn*26*). (B) *Acinetobacter baylyi* ADP1 parental and mutant populations exposed to 15 µM ethidium bromide. The ADP1 Δ*adeN* mutant (*A. baylyi* CM202) was randomly selected on chloramphenicol selective medium (5) and contains a single nucleotide deletion in the center of the gene (the region encoding the sixth α-helix of AdeN), resulting in a frameshift. The Δ*adeJ* mutant was constructed by allelic replacement, where the entire gene was replaced with a kanamycin resistance cassette (5). Each curve shows the fluorescence intensity of 100,000 cells. The cell populations show distinct fluorescence profiles, based on the concentration of ethidium in the cell cytoplasm. Download Figure S1, JPG file, 0.1 MB

Figure S2  Gating during flow cytometry to examine only single *A. baumannii* cells of uniform size. (A) Single particles were gated based on forward scatter and the forward scatter trigger pulse width (red gate labeled “singles”). (B) Cells within this gate displaying uniform side scatter were selected as single living cells (green gate labeled “cells”) in which total fluorescence was likely to reflect the internal ethidium concentration. Download Figure S2, JPG file, 0.2 MB

Figure S3  Genes negatively selected by flow sorting. Bars represent the fold change in mutant abundance in cells selected for low ethidium fluorescence (blue), high ethidium fluorescence (red), or growth in 62.5 µg/ml (approximately 158 µM) ethidium bromide (hatched green; 1/4× MIC), compared to the starting mutant pool. Positive values indicate higher mutant abundance in the selected pool, whereas negative values indicate lower abundance. For all genes shown, significantly negative fold changes were observed in both flow-sorted mutant pools (>2-fold change, *Q* value of <0.05). Asterisks indicate fold change values for the ethidium bromide chemically selected pool that are greater than 2-fold and supported by a *Q* value of 0.05 or below. Download Figure S3, JPG file, 0.3 MB

Figure S4  Genes positively selected by flow sorting. Bars represent the fold change in mutant abundance in cells selected for low ethidium fluorescence (blue), high ethidium fluorescence (red), or growth in 62.5 µg/ml (approximately 158 µM) ethidium bromide (hatched green; 1/4× MIC), compared to the starting mutant pool. Positive values indicate higher mutant abundance in the selected pool, whereas negative values indicate lower abundance. For all genes shown, significantly positive fold changes were observed in both flow-sorted mutant pools (>2-fold change, *Q* value of <0.05). Asterisks indicate fold change values for the ethidium bromide chemically selected pool that are greater than 2-fold and supported by a *Q* value of 0.05 or below. Download Figure S4, JPG file, 0.2 MB

Figure S5  Distribution of insertion indexes for genes annotated in the *A. baumannii* BAL062 genome. The insertion index for each gene was calculated by dividing the number of unique insertions within the first 90% of the gene by the gene length. Bars represent the number of genes (density) with a particular insertion index. The indexes show a bimodal distribution corresponding to genes that are able to tolerate insertions and those that are not able to tolerate insertions when grown under permissive conditions in MH broth. Genes with an insertion index below 0.0047 (*n* = 475; red line), including 350 genes for which no transposon insertion mutants were detected, were predicted to be essential and were excluded from the analyses. Download Figure S5, JPG file, 0.1 MB

Data Set S1 Microsoft Excel file showing the log_2_ fold changes in mutant abundance between the FACS-selected or ethidium-selected mutant pools and the control mutant pools and *Q* values for all genes examined in this study. Download Data Set S1, XLSX file, 0.5 MB

Table S1  Genes encoding putative efflux pumps in *A. baumannii* BAL062 and their level of selection by FACS or ethidium. Genes were identified using the Transporter Automated Annotation Pipeline (TransAAP; http://www.membranetransport.org). The log_2_ fold changes in mutant abundance between the FACS-selected or ethidium-selected mutant pools and the control mutant pools and *Q* values are shown for each putative efflux gene. Table S1, DOCX file, 0.2 MB

Table S2  Sequence read numbers, insertion counts, and European Nucleotide Archive accession numbers for the TraDIS replicate samples sequenced in this study. Table S2, DOCX file, 0.1 MB

Table S3  Number of particles analyzed and numbers of cells collected in each replicate TraDISort FACS experiment. Table S3, DOCX file, 0.1 MB
