# Supplementary figures and images for: Fluorescence-Based Flow Sorting in Parallel with Transposon Insertion Site Sequencing Identifies Multidrug Efflux Systems in Acinetobacter baumannii
Source: mBio. 2016 Sep 6;7(5):e01200-16. doi: 10.1128/mBio.01200-16 (PMC5013296; doi:10.1128/mBio.01200-16)

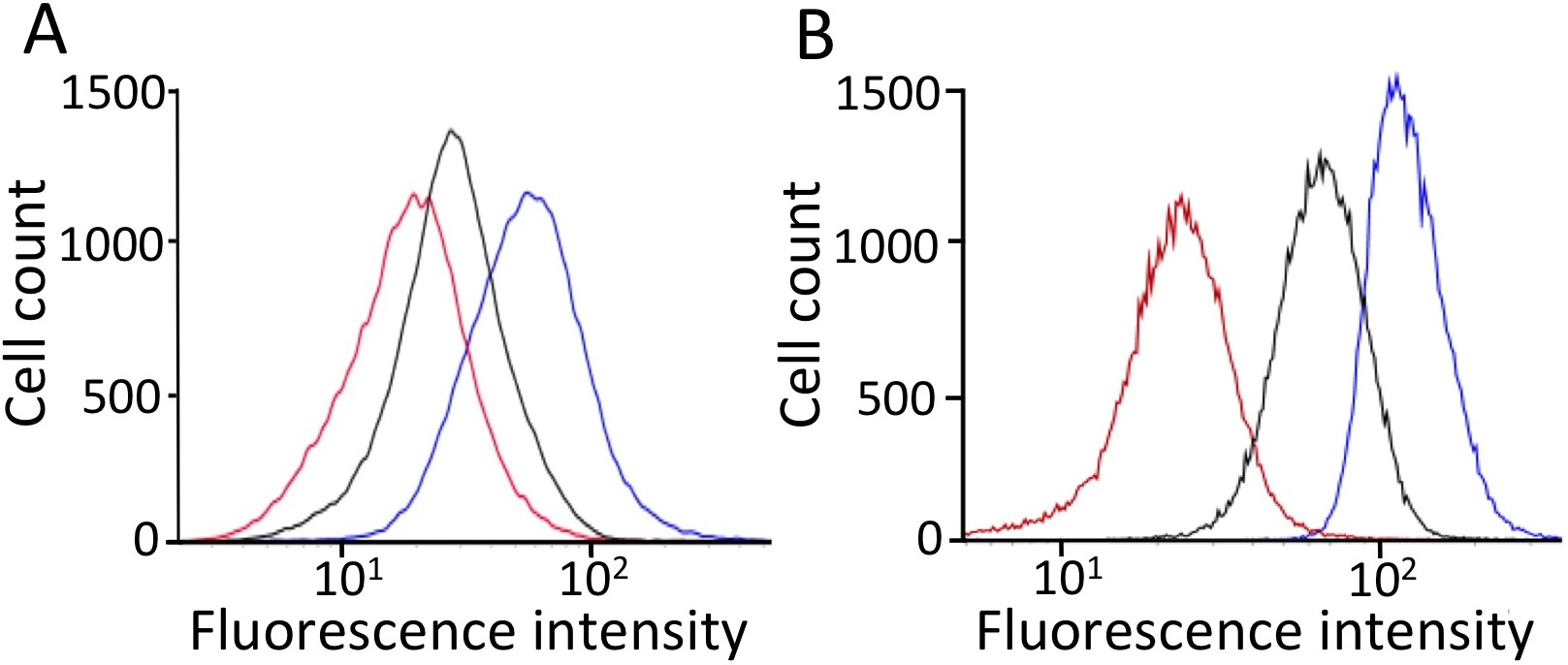

Supplement: Figure S1 — Flow cytometric analysis of Acinetobacter parental (black), and ΔadeN (red) and ΔadeJ (blue) mutant populations exposed to ethidium bromide. (A) A. baumannii AB5075-UW parental and mutant populations exposed to 40 µM ethidium bromide. The AB5075-UW strains were obtained from the Manoil lab collection (9). The mutants carry Tn26 insertions in adeJ (ABUW_0843-122::Tn26) and adeN (ABUW_1731-148::Tn26). (B) Acinetobacter baylyi ADP1 parental and mutant populations exposed to 15 µM ethidium bromide. The ADP1 ΔadeN mutant (A. baylyi CM202) was randomly selected on chloramphenicol selective medium (5) and contains a single nucleotide deletion in the center of the gene (the region encoding the sixth α-helix of AdeN), resulting in a frameshift. The ΔadeJ mutant was constructed by allelic replacement, where the entire gene was replaced with a kanamycin resistance cassette (5). Each curve shows the fluorescence intensity of 100,000 cells. The cell populations show distinct fluorescence profiles, based on the concentration of ethidium in the cell cytoplasm. Download [file mbo004162978sf1.jpg]

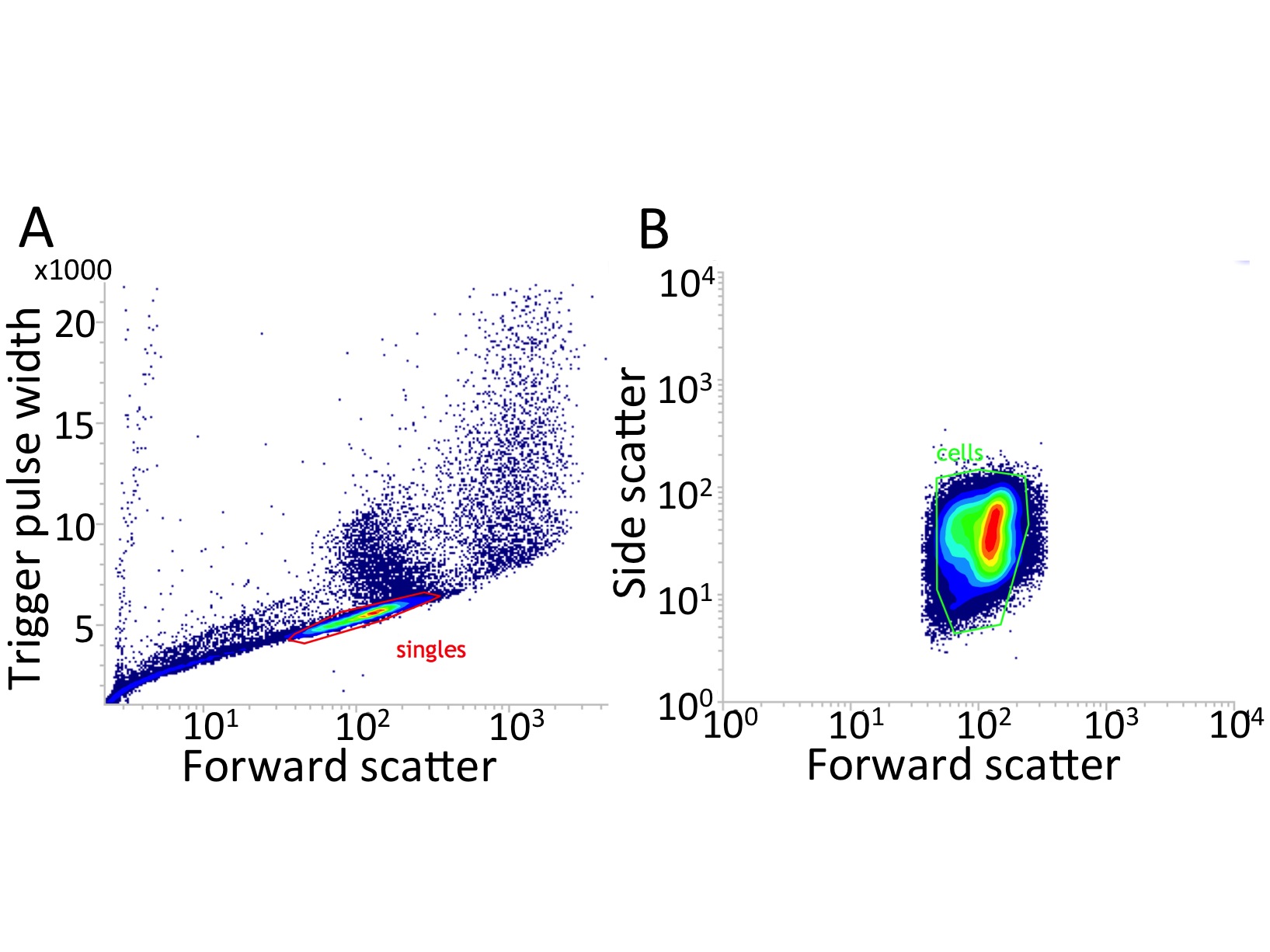

Supplement: Figure S2 — Gating during flow cytometry to examine only single A. baumannii cells of uniform size. (A) Single particles were gated based on forward scatter and the forward scatter trigger pulse width (red gate labeled “singles”). (B) Cells within this gate displaying uniform side scatter were selected as single living cells (green gate labeled “cells”) in which total fluorescence was likely to reflect the internal ethidium concentration. Download [file mbo004162978sf2.jpg]

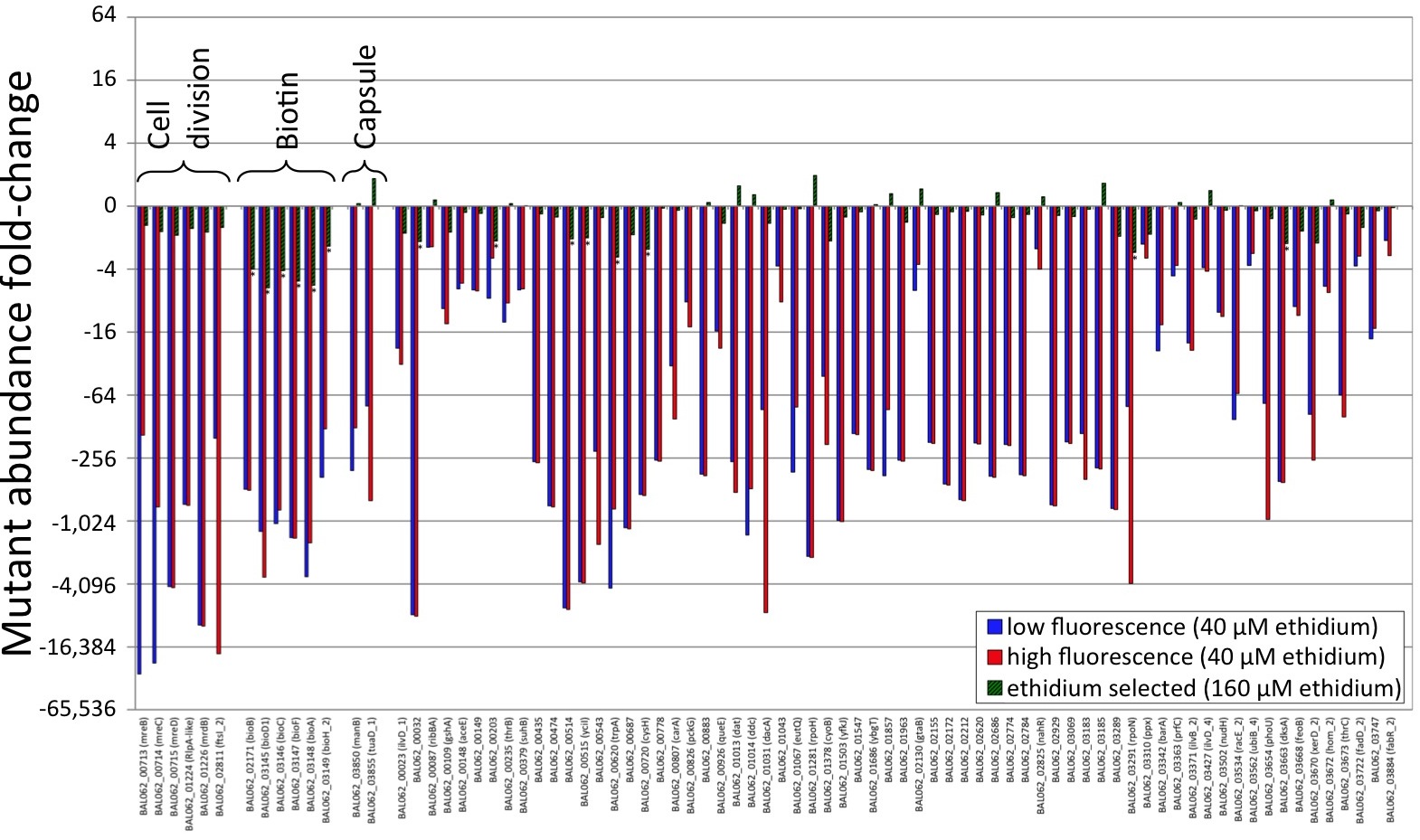

Supplement: Figure S3 — Genes negatively selected by flow sorting. Bars represent the fold change in mutant abundance in cells selected for low ethidium fluorescence (blue), high ethidium fluorescence (red), or growth in 62.5 µg/ml (approximately 158 µM) ethidium bromide (hatched green; 1/4× MIC), compared to the starting mutant pool. Positive values indicate higher mutant abundance in the selected pool, whereas negative values indicate lower abundance. For all genes shown, significantly negative fold changes were observed in both flow-sorted mutant pools (>2-fold change, Q value of <0.05). Asterisks indicate fold change values for the ethidium bromide chemically selected pool that are greater than 2-fold and supported by a Q value of 0.05 or below. Download [file mbo004162978sf3.jpg]

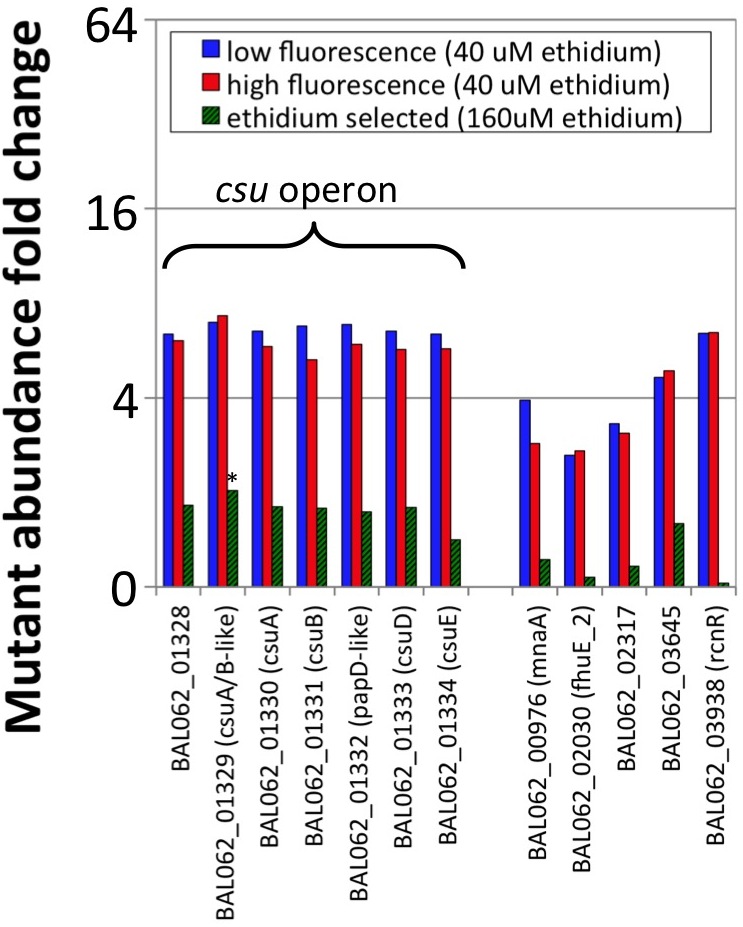

Supplement: Figure S4 — Genes positively selected by flow sorting. Bars represent the fold change in mutant abundance in cells selected for low ethidium fluorescence (blue), high ethidium fluorescence (red), or growth in 62.5 µg/ml (approximately 158 µM) ethidium bromide (hatched green; 1/4× MIC), compared to the starting mutant pool. Positive values indicate higher mutant abundance in the selected pool, whereas negative values indicate lower abundance. For all genes shown, significantly positive fold changes were observed in both flow-sorted mutant pools (>2-fold change, Q value of <0.05). Asterisks indicate fold change values for the ethidium bromide chemically selected pool that are greater than 2-fold and supported by a Q value of 0.05 or below. Download [file mbo004162978sf4.jpg]

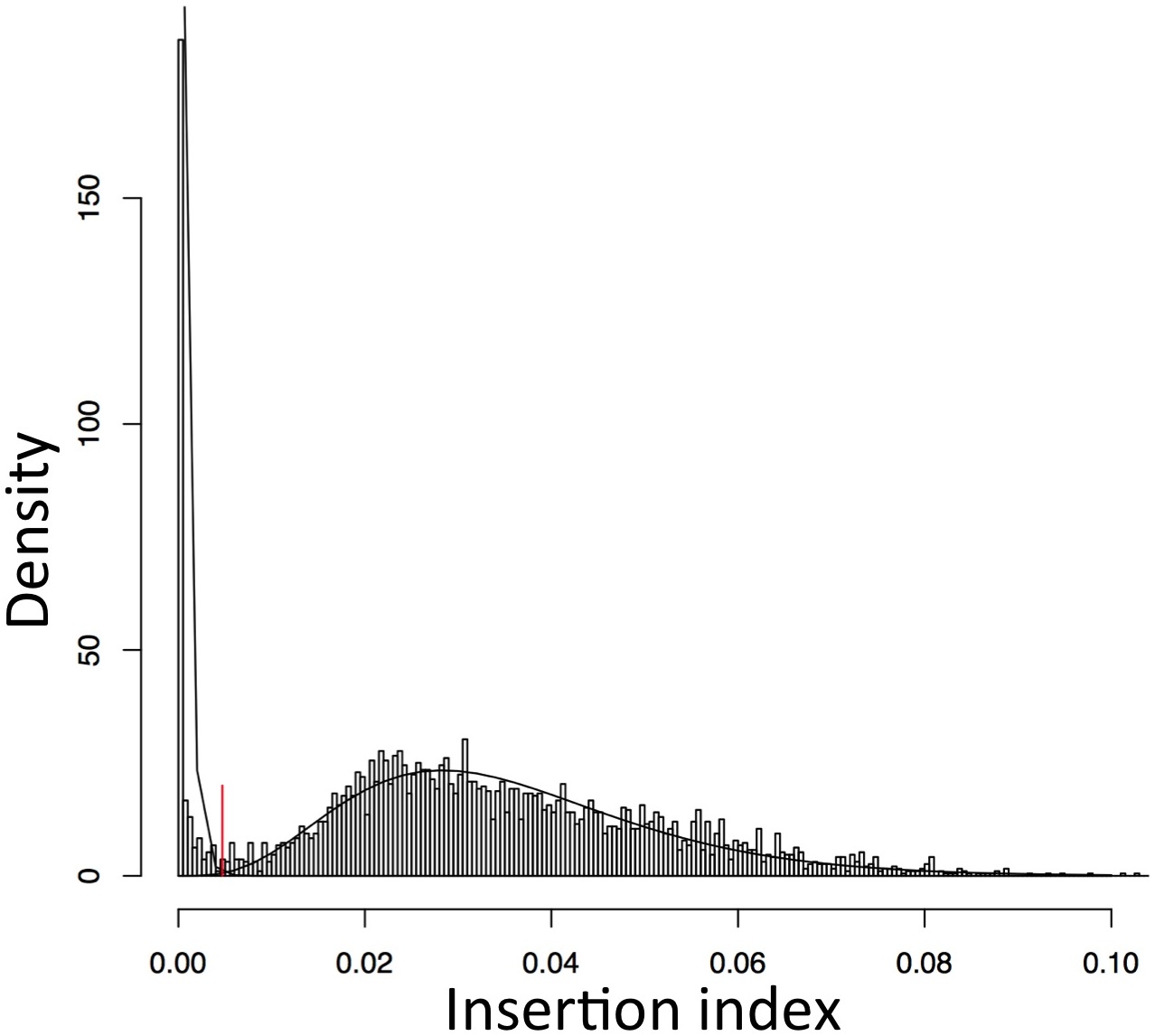

Supplement: Figure S5 — Distribution of insertion indexes for genes annotated in the A. baumannii BAL062 genome. The insertion index for each gene was calculated by dividing the number of unique insertions within the first 90% of the gene by the gene length. Bars represent the number of genes (density) with a particular insertion index. The indexes show a bimodal distribution corresponding to genes that are able to tolerate insertions and those that are not able to tolerate insertions when grown under permissive conditions in MH broth. Genes with an insertion index below 0.0047 (n = 475; red line), including 350 genes for which no transposon insertion mutants were detected, were predicted to be essential and were excluded from the analyses. Download [file mbo004162978sf5.jpg]
