# Supplementary material for: Fluorescence-Based Flow Sorting in Parallel with Transposon Insertion Site Sequencing Identifies Multidrug Efflux Systems in Acinetobacter baumannii
Source: mBio. 2016 Sep 6;7(5):e01200-16. doi: 10.1128/mBio.01200-16 (PMC5013296; doi:10.1128/mBio.01200-16)
Supplement: Table S1 — Genes encoding putative efflux pumps in A. baumannii BAL062 and their level of selection by FACS or ethidium. Genes were identified using the Transporter Automated Annotation Pipeline (TransAAP; http://www.membranetransport.org). The log2 fold changes in mutant abundance between the FACS-selected or ethidium-selected mutant pools and the control mutant pools and Q values are shown for each putative efflux gene. [file mbo004162978st1.docx]

Table S1. Genes encoding putative efflux pumps in *A. baumannii* BAL062 and their level of selection by FACS or ethidium.

| **locus_tag*** | **low (logFC)** | **low (q.value)** | **high (logFC)** | **high (q.value)** | **Et (logFC)** | **Et (q.value)** |
| --- | --- | --- | --- | --- | --- | --- |
| BAL062_00007 | -0.2766 | 0.9052 | 0.3279 | 0.9998 | -0.3206 | 0.0128 |
| BAL062_00031 | -0.3198 | 0.7578 | -0.3209 | 0.9998 | -0.2002 | 0.2149 |
| BAL062_00037 | 0.2935 | 0.9143 | -1.0603 | 0.9998 | -0.2498 | 0.0927 |
| BAL062_00060 | 0.1154 | 0.9818 | -0.3510 | 0.9998 | -0.0262 | 0.9251 |
| BAL062_00104 | -0.0054 | 0.9997 | -0.0939 | 0.9998 | -0.0211 | 0.9440 |
| BAL062_00191 | -0.5228 | 0.7180 | -0.5067 | 0.9998 | 0.1255 | 0.4947 |
| BAL062_00208 | 0.0053 | 0.9997 | 0.3960 | 0.9998 | 0.1933 | 0.2857 |
| BAL062_00644 | 0.0755 | 0.9936 | 0.0575 | 0.9998 | -0.0432 | 0.8878 |
| BAL062_00674 | 0.3059 | 0.8565 | 0.1773 | 0.9998 | 0.0266 | 0.9218 |
| BAL062_00698 | 0.1223 | 0.9818 | -0.5763 | 0.9998 | 0.0761 | 0.7599 |
| BAL062_00823 | 0.5519 | 0.4957 | 0.2115 | 0.9998 | 0.0773 | 0.6880 |
| BAL062_00835 | 0.0949 | 0.9846 | 0.1495 | 0.9998 | -0.0477 | 0.8648 |
| BAL062_00875 | -0.3693 | 0.9613 | -0.5886 | 0.9998 | 0.1259 | 0.5585 |
| BAL062_00916 | -0.0004 | 1.0000 | 0.0422 | 0.9998 | 0.1057 | 0.6794 |
| BAL062_00992 | 0.2849 | 0.9627 | 0.3680 | 0.9998 | 0.0460 | 0.8624 |
| BAL062_01073 | 0.2067 | 0.9903 | -0.7696 | 0.9998 | 0.0517 | 0.8624 |
| BAL062_01229 | -0.7220 | 0.9203 | -2.0534 | 0.9998 | -0.1456 | 0.5453 |
| BAL062_01230 | -0.2105 | 0.9818 | 0.1625 | 0.9998 | -0.0851 | 0.7361 |
| BAL062_01336 | 0.4197 | 0.9143 | 0.0254 | 0.9998 | -0.0273 | 0.9292 |
| BAL062_01350 | 0.6115 | 0.8497 | 0.5384 | 0.9998 | -0.0419 | 0.9079 |
| BAL062_01414 | -0.1922 | 0.9725 | 0.3359 | 0.9998 | -0.0050 | 0.9890 |
| BAL062_01421 | 0.5632 | 0.9047 | 0.6820 | 0.9998 | -0.1070 | 0.6546 |
| BAL062_01586 | 0.6407 | 0.8815 | 0.3031 | 0.9998 | 0.0911 | 0.7093 |
| BAL062_01824 | 0.8751 | 0.7209 | -0.8070 | 0.9998 | 0.0376 | 0.9101 |
| BAL062_01835 | -0.5129 | 0.9247 | -1.3068 | 0.9998 | 0.0320 | 0.9194 |
| BAL062_01836 | 1.1995 | 0.0222 | 0.5034 | 0.9998 | -0.0285 | 0.9500 |
| BAL062_01872 | -0.0959 | 0.9997 | 0.3713 | 0.9998 | -0.1217 | 0.6809 |
| BAL062_01873 | 0.0849 | 0.9997 | 1.2837 | 0.9998 | 0.0262 | 0.9343 |
| BAL062_01967 | 0.8450 | 0.9527 | -1.0098 | 0.9998 | -0.2031 | 0.5430 |
| BAL062_02024 | 1.2499 | 0.8063 | 0.6576 | 0.9998 | 0.1026 | 0.8375 |
| BAL062_02025 | 0.8482 | 0.9211 | 0.2319 | 0.9998 | -0.1061 | 0.8301 |
| BAL062_02026 | 0.9488 | 0.6569 | -0.5321 | 0.9998 | 0.0748 | 0.8129 |
| BAL062_02052 | 0.2479 | 0.9818 | 1.2810 | 0.5942 | -0.0497 | 0.8655 |
| BAL062_02145 | 0.4578 | 0.9195 | 0.6159 | 0.9998 | -0.0395 | 0.8836 |
| BAL062_02249 | 0.5249 | 0.9047 | 0.4401 | 0.9998 | 0.0658 | 0.8473 |
| BAL062_02267 | 0.2362 | 0.9845 | 0.3434 | 0.9998 | -0.0105 | 0.9799 |
| BAL062_02327 | 0.2643 | 0.9867 | -0.3628 | 0.9998 | -0.0633 | 0.8639 |
| BAL062_02338 | -2.1146 | 0.7721 | -0.0698 | 0.9998 | 0.0136 | 0.9757 |
| BAL062_02443 | 0.1625 | 0.9807 | 0.6356 | 0.9998 | 0.0357 | 0.8995 |
| BAL062_02520 | 0.6024 | 0.8773 | 0.7981 | 0.9463 | 0.0392 | 0.8735 |
| BAL062_02611 | 1.2659 | 0.8874 | -2.0945 | 0.9998 | -0.0687 | 0.8952 |
| BAL062_02873 | -0.0124 | 0.9997 | -0.0912 | 0.9998 | 0.1409 | 0.4491 |
| BAL062_03044 | 0.4457 | 0.6757 | 0.5297 | 0.9998 | 0.0587 | 0.8255 |
| BAL062_03179 | -0.6804 | 0.3536 | 0.1903 | 0.9998 | -0.1845 | 0.2861 |
| BAL062_03180 | 0.1618 | 0.9725 | 0.3500 | 0.9998 | -0.0728 | 0.7592 |
| BAL062_03245 | -0.1386 | 0.9818 | 0.2478 | 0.9998 | -0.1041 | 0.5867 |
| BAL062_03263 | 0.2737 | 0.9725 | -0.5232 | 0.9998 | -0.1189 | 0.5874 |
| BAL062_03322 | 0.0735 | 0.9903 | -0.1744 | 0.9998 | -0.2170 | 0.1391 |
| BAL062_03353 | -0.1500 | 0.9811 | -0.3355 | 0.9998 | -0.0715 | 0.7774 |
| BAL062_03376 | 0.1231 | 0.9867 | 0.4847 | 0.9998 | 0.0559 | 0.7976 |
| BAL062_03394 | 0.0178 | 0.9997 | 0.3957 | 0.9998 | -0.1421 | 0.4727 |
| BAL062_03729 | -0.5917 | 0.4560 | -0.0561 | 0.9998 | -0.1869 | 0.2861 |
| BAL062_03755 | -0.0222 | 0.9997 | -0.0471 | 0.9998 | -0.2253 | 0.1584 |
| BAL062_03797 | -0.0070 | 0.9997 | -0.0660 | 0.9998 | -0.0524 | 0.8375 |
| BAL062_03812 | -0.1374 | 0.9725 | 0.1606 | 0.9998 | -0.0704 | 0.7695 |
| BAL062_03832 | 0.1998 | 0.9317 | -0.1097 | 0.9998 | -0.0522 | 0.8518 |

* Genes were identified using the Transporter Automated Annotation Pipeline (TransAAP; www.membranetransport.org). The numbers show the log2 fold-change in abundance of transposon insertion mutants in each gene between the FACS selected or ethidium selected mutant pools compared to the control pools, and the Q-value.
