# Supplementary material for: Fluorescence-Based Flow Sorting in Parallel with Transposon Insertion Site Sequencing Identifies Multidrug Efflux Systems in Acinetobacter baumannii
Source: mBio. 2016 Sep 6;7(5):e01200-16. doi: 10.1128/mBio.01200-16 (PMC5013296; doi:10.1128/mBio.01200-16)
Supplement: Table S2 — Sequence read numbers, insertion counts, and European Nucleotide Archive accession numbers for the TraDIS replicate samples sequenced in this study. [file mbo004162978st2.docx]

Table S2. Sequence read and insertion counts, and accession numbers

|  | **Lane 1** | | | **Lane 2** | | |
| --- | --- | --- | --- | --- | --- | --- |
| **Sample name** | **Total Reads** | **Total Unique Insertion Sites** | **ENA Accession*** | **Total Reads** | **Total Unique Insertion Sites** | **ENA Accession** |
| **MH control 1** | 4,515,158 | 105,598 | **ERR1203846** | 4,507,871 | 106,085 | **ERR1203876** |
| **MH control 2** | 5,011,255 | 106,798 | **ERR1203847** | 4,999,533 | 107,303 | **ERR1203877** |
| **Ethidium selected 1** | 4,719,043 | 113,506 | **ERR1288990** | 4,751,070 | 110,800 | **ERR1289030** |
| **Ethidium selected 2** | 4,083,597 | 109,850 | **ERR1288991** | 4,118,277 | 107,840 | **ERR1289031** |
| **Low fluorescence 2** | 3,733,354 | 29,100 | **ERR1288992** | 3,740,728 | 26,820 | **ERR1289032** |
| **High fluorescence 2** | 4,366,882 | 27,527 | **ERR1288993** | 4,396,165 | 25,410 | **ERR1289033** |
| **Low fluorescence 3** | 3,917,835 | 29,401 | **ERR1288994** | 3,943,891 | 27,058 | **ERR1289034** |
| **High fluorescence 3** | 4,589,754 | 24,596 | **ERR1288995** | 4,624,915 | 22,374 | **ERR1289035** |
| **Low fluorescence 4** | 3,902,428 | 36,853 | **ERR1288996** | 3,931,909 | 34,531 | **ERR1289036** |
| **High fluorescence 4** | 4,558,555 | 29,189 | **ERR1288997** | 4,588,395 | 26,414 | **ERR1289037** |
| **Low fluorescence 1** | 4,497,450 | 22,434 | **ERR1288998** | 4,532,821 | 20,226 | **ERR1289038** |
| **High fluorescence 1** | 3,968,412 | 16,888 | **ERR1288999** | 3,995,713 | 15,066 | **ERR1289039** |

* Sequence reads deposited in the European Nucleotide Archive.
