# Supplementary material for: Fluorescence-Based Flow Sorting in Parallel with Transposon Insertion Site Sequencing Identifies Multidrug Efflux Systems in Acinetobacter baumannii
Source: mBio. 2016 Sep 6;7(5):e01200-16. doi: 10.1128/mBio.01200-16 (PMC5013296; doi:10.1128/mBio.01200-16)
Supplement: Table S3 — Number of particles analyzed and numbers of cells collected in each replicate TraDISort FACS experiment. [file mbo004162978st3.docx]

Table S3. Cell counts from TraDIS library FACS experiments

| **Sort number** | **Total events (total cells screened)** | **Low fluorescent cell count** | **High fluorescent cell count** |
| --- | --- | --- | --- |
| 1 | 3,904,416 | 25,322 | 21,780 |
| 2 | 9,719,120 | 45,409 | 41,400 |
| 3 | 10,577,929 | 40,529 | 40,038 |
| 4 | 13,101,390 | 63,997 | 50,025 |
| **Total** | **37,302,855** | **175,257** | **153,243** |
